# SUMOylation of EphB4 enhances its stability in prostate cancer

**DOI:** 10.1038/s41416-026-03442-w

**Published:** 2026-04-15

**Authors:** Mohanan Sada Nand Maharaj, Inga Mertens-Walker, Jessica E. Lisle, Adrian Herington, Carson Stephens, Melissa Chai, Wim Meutermans, Sally-Anne Stephenson

**Affiliations:** 1 Centre for Genomics and Personalised Health, School of Biomedical Sciences, Faculty of Health, Queensland University of Technology, Translational Research Institute, Woolloongabba, QLD Australia; 2 School of Biomedical Sciences, Faculty of Health, Queensland University of Technology, Translational Research Institute, Woolloongabba, QLD Australia

**Keywords:** Prostate cancer, Sumoylation

## Abstract

**Background:**

The receptor tyrosine kinase EphB4 is frequently overexpressed in epithelial cancers, including prostate cancer (PCa). SUMOylation is a post-translational modification that influences protein interactions, localisation and stability. This study investigated how SUMOylation regulates EphB4 localisation, stability and function in PCa.

**Methods:**

EphB4 SUMOylation was analysed in PCa cell lines and its contribution to stability assessed using siRNA or chemical inhibition of SUMOylation. An EphB4 mutant protein (K616R) was expressed in PCa cells and proteasomal inhibition was used to assess its stability. Cell migration was measured using a scratch wound assay. Immunoprecipitation was used to determine if mutant EphB4 could be SUMOylated on other residues.

**Results:**

EphB4 in PCa cells is constitutively modified by SUMO2/3 and acquires SUMO1 modification when stimulated with ephrin-B2 ligand. SUMOylation of K616 is critical for EphB4 stability. K616R EphB4 protein is degraded by the proteasome, and this is associated with reduced MYC protein and slower migration. Immunoprecipitation revealed that additional SUMOylated lysines may also contribute to EphB4 function.

**Conclusions:**

SUMOylation at K616 stabilises EphB4, promotes MYC signalling and PCa cell migration. These findings identify a novel mechanism regulating EphB4 and highlight SUMOylation as a potential target in EphB4-driven cancers.

## Introduction

In 2022, prostate cancer was estimated to become the fourth most common cancer worldwide and the second most common cancer in men, with more than 1.4 million new cases diagnosed [1]. The 14 Erythropoietin-producing hepatocellular (Eph) receptors constitute the largest subfamily of receptor tyrosine kinases (RTKs) [2]. Over-expression, loss of cell-cell contacts and Eph mis-localisation facilitate non-canonical signalling that has been widely associated with tumour promotion [3]. Several Eph receptors have been linked to prostate cancer, including EphB4, which is overexpressed in 66% of prostate cancer tissues, where it contributes to signalling pathways associated with viability, migration and invasion [4–9]. EphB4 expression appears to be induced by loss of tumour suppressors p53 and PTEN, inactivating mutations of which are also linked to prostate cancer initiation [7, 8]. EphB4 expression is also influenced by the PI3K signalling pathway [9] while exogenous over-expression of EphB4 in established prostate cancer cell lines leads to a more aggressive phenotype [10].

Knockdown experiments using in vivo PC3 xenografts show that EphB4 is required for the continued growth of established tumours [7]. More recently, EphB4 inhibition has been linked to activation of cell death pathways that also increased cell surface localisation of calreticulin, and release of HMGB1 and ATP, all changes associated with immunogenic cell death, suggesting an effective treatment for EphB4-positive tumours might be achieved by combining EphB4-targeted drugs with an immune checkpoint blockade [8]. An array of compounds, including small molecule kinase inhibitors, antibodies and both ligand mimetic and antagonistic peptides, have been developed, but the role of EphB4 in cancer is complicated with receptor clustering, bidirectional signalling having both tumour-suppressing and tumour-promoting activities and functions that are ligand-dependent, ligand-independent, kinase-dependent and kinase-independent [2, 8–12].

SUMOylation is the covalent addition of a small ubiquitin-like modifier (SUMO) protein to a target protein *via* an isopeptide bond formed between the C-terminal glycine of SUMO and the ε-amino group of a specific lysine of the target [13]. SUMOylation changes protein-protein interactions, cellular localisation (cytoplasmic to nuclear translocation), protein function and protein stability [14]. SUMOylation is a reported modification of seven other RTKs (EphB1, EGFR, IGF-IR, ErbB4, PDGFRα, VEGFR2 and FGFR1) [15–24]. Chen et al. (2018) found that EphB1 exogenously over-expressed in HEK293T cells is modified with SUMO1 at K785 and this suppresses the activation of PKCγ, resulting in tumour suppression [15]. EGF stimulation induces SUMOylation of EGFR at K37, causing translocation to the nucleus [16]. IGF-1 stimulation promotes the SUMO1 modification of IGF-1R on K1025, K1100 and K1120 [17]. Although this has no effect on kinase activity, it is required for receptor translocation to the nucleus and increases IGF-1R stability, allowing it to accumulate in the nucleus to influence gene expression [17, 18]. Ligand binding to ErbB4 activates regulated intramembrane proteolysis (RIP), releasing an intracellular domain (ICD) that translocates to the nucleus, where it co-localises with E3 ligase PIAS3, SUMO-1 and PML [19]. The lysine of the ErbB4 ICD to which SUMO-1 is conjugated was identified as K714 [20]. SUMOylation of PDGFRα on K917 was linked to activation of PLC-γ, decreased activation of STAT3 and increased proliferation in response to PDGF-BB stimulation [21]. He et al. (2023) report that in endothelial cells, VEGFR2 is SUMOylated on K1270 and accumulates in the Golgi [22]. On stimulation, VEGFR2 is de-SUMOylated by SENP6 and moved to the cell membrane, increasing angiogenesis [22, 23]. FGFR1 is also SUMOylated constitutively in endothelial cells in response to proangiogenic stimulation and facilitates its interaction with FRS2α to regulate VEGFR2 activity in angiogenesis [24].

We have previously reported the nuclear localisation of EphB4 in cancer cells and that EphB4 has two functional nuclear localisation sequences [25]. Given the recent discoveries linking SUMOylation to nuclear localisation of several RTKs [16–20], we explored whether EphB4 was also SUMOylated in cancer cells. Here we show that EphB4 is SUMOylated on K616, a lysine residue in the N lobe of the kinase domain and within a consensus motif predicted with high probability. We establish that EphB4 SUMOylation contributes to the stability of the receptor, as the receptor is degraded via the proteasome if it cannot be SUMOylated on lysine 616, providing a mechanism for its over-expression in prostate cancer. Further, we demonstrate that EphB4 SUMOylation is linked to the expression of *c-myc* and has a pro-migratory function in prostate cancer cells.

## Methods

### Cell lines and culture conditions

Human prostate cancer cell lines PC-3, LNCaP, DU145, 22Rv1 and the renal cancer cell line 786-0, were purchased from the American Type Culture Collection (ATCC) (Manassas, VA, USA) and cultured in HEPES-buffered RPMI 1640 medium (Thermo Scientific, Queensland, Australia), pH 7.4, supplemented with 10% foetal calf serum (FCS) (Thermo Scientific) in vented tissue culture flasks and a humidified incubator at 37 °C and 5% CO_2_. Breast cancer cell lines MDA-MB-231, MCF7 and BT474, the colon cancer cell line HT29 and HEK293T cells were also purchased from the American Type Culture Collection and cultured in DMEM (Thermo Scientific) supplemented with 10% FCS. MCF10A was cultured in DMEM/F12 (Thermo) supplemented with 5% Horse Serum (Thermo), 20 ng/ml EGF (Sigma Aldrich/Merck, Melbourne, Australia), 0.5 mg/ml hydrocortisone (Sigma Aldrich/Merck), 100 ng/ml cholera toxin (Sigma Aldrich/Merck) and 10 µg/ml insulin (Sigma Aldrich/Merck). All cell lines were confirmed to be free of mycoplasma and had been validated by STR profiling. Approval to use the cancer cell lines was gained from the Queensland University of Technology Human Research Ethics Committee.

### Antibodies

Antibodies purchased from Cell Signalling Technology (New England Biolabs, Victoria, Australia) include primary antibodies specific to EphB4 (14960), Ubc9 (4918), PLK1 (4513), MYC (9402) and p-P38 MAPK (4511). Antibodies purchased from Santa Cruz Biotechnology (Dallas, Texas) include GAPDH (SC-47724) and RanGAP1 (SC-28322). Antibodies from Thermo include EphB4 (3D7G9 – Zymed), RFP (R10367) and α-Tubulin (62204). Antibodies purchased from Novus Biologicals (Centennial, CO, USA) include SUMO2/3 (SPM572) and GFP (600308). The SUMO1 (AM1200a) antibody was purchased from Abcepta (San Diego, CA, USA). C2 and 6H4 EphB4 antibodies were created and validated in-house [26]. Secondary antibodies for Western analysis included IRDye 680RD donkey anti-rabbit (LI-COR, cat#926-68073) and IRDye 800CW donkey anti-mouse (LI-COR, cat#926-32212).

### Ligand stimulation and Immunofluorescence

Cells were cultured in flasks or plates for 48 h before they were serum starved (0.1% serum for 2-16 h) and then stimulated for 15 min with 2 μg/ml soluble ligand ephrin-B2/Fc (R&D Systems, Minneapolis, MN, USA) pre-clustered for 1 h with AffiniPure goat anti-human IgG, Fcγ (Jackson ImmunoResearch, West Grove, PA). As a negative control, cells were treated with the Fc fragment alone, also pre-clustered in the same way (α-Fc). For immunofluorescence staining, cells were cultured in 8-well glass chamber slides for 48 h, serum starved for 2 h, then stimulated with clustered ephrin-B2/Fc ligand or control clustered Fc alone, then washed with PBS, and fixed with 4% paraformaldehyde for 10 min. After permeabilization with 0.3% Triton X-100 for 3 min, non-specific binding was blocked with 10% goat serum for 1 h. Fixed cells were incubated overnight at 4 °C with primary antibodies diluted 1:1000 in 10% goat serum, with unbound antibody then removed using three PBS washes. Secondary antibodies (goat anti-mouse Alexa Fluor^TM^ 488 and goat anti-rabbit Alexa Fluor^TM^ 562) were then added for 1 h at room temperature, unbound antibody removed with three PBS washes and cells then stained with 0.3 µM DAPI in PBS for 10 min. After three more PBS washes, slides were mounted with Mowiol® 4-88 (Sigma Aldrich/Merck) and coverslips applied. Fluorescence was visualised using a Nikon Spinning Disk Confocal Microscope.

### Generating over-expressing cells

The complete EphB4 coding sequence (without the stop codon) was subcloned from pIRES-neo2 [10] into pENTR1A-GFP-N2 (Addgene 19364) in frame with the eGFP tag. SUMO1, SUMO2 and SUMO3 coding sequences were cloned using the *Age*I and *Xho*I restriction sites into the pENTR-mScarlet-i vector (unpublished, a kind gift from Dr Robert Ju, University of Queensland, Australia). pENTR constructs were recombined with pLEX_307 (Addgene #41392) for 2 h at 25 °C using Gateway® LR Clonase® Enzyme (Thermo). The reaction was inactivated with Proteinase K (Thermo) at 37 °C for 10 min. Lentivirus was produced by co-transfecting HEK293FT cells with the Rev plasmid (0.5 µg), envelope plasmid (2.8 µg pVSV-G), packaging plasmid (7.1 µg pNHP), and lentiviral construct (3.5 µg pLEX_307) using Lipofectamine 3000. After 48 h, the viral supernatant was collected and used to transduce target cells for 24 h, followed by medium replacement and puromycin selection (1 µg/ml) to create stable polyclonal cell lines.

### Site-directed mutagenesis

Site-directed mutagenesis was used to create mutant constructs with lysine-to-arginine substitutions at position K616. The primers for the K616R mutation were: forward, 5’-GATCGATGTCTCCTACGTCAGGATTGAAGAGGTGATTGGTG-3’; reverse, 5’-CACCAATCACCTCTTCAATCCTGACGTAGGAGACATCGATC-3’. Mutagenesis was performed using Pfu Ultra II Fusion HS DNA Polymerase (Agilent Technologies, Santa Clara, CA, USA) according to the manufacturer’s instructions. PCR reactions were carried out with 50 ng pENTR1A-EphB4-GFP-N2 template, 2.5 µl 10X PCR buffer, 1 µl 10 mM dNTPs, 1 µl 10 µM primers, and 0.5 µl polymerase. The cycling conditions were: 95 °C for 2 min, 20 cycles of 95 °C for 2 min, 68 °C for 30 sec, 72 °C for 7 min + 4 sec per cycle, and final extension at 72 °C for 10 min. After cycling, the template DNA was digested with *Dpn*I for 2 h at 37 °C, followed by heat inactivation. *E. coli* JM109 cells were heat-shock transformed with the digested reaction, and colonies were selected on LB agar plates with 50 ng/ml kanamycin sulfate.

### Preparation of protein lysates

The tissue culture flask or plate was placed on ice, the medium removed and the cells washed with ice-cold PBS. Cells were then lysed with pre-cooled Lysis Buffer (RIPA buffer (Thermo), 1% HALT protease and phosphatase inhibitor (Thermo), 1% EDTA (Thermo), 20 mM N-Ethylmaleimide (Sigma)) by rocking at 4 °C for 20 min. The lysates were collected using a cell scraper and removed from the flask to a 1.5 mL Eppendorf tube. Genomic DNA was disrupted with a 29-gauge needle, and the sample was sonicated for 5 min at 4 °C. After centrifugation at 14,000 rpm for 30 min to remove the insoluble fraction, soluble protein concentration was measured using the BCA Protein Assay Kit (Thermo) with BSA standards.

### Western analysis

Protein samples (10–20 µg) were loaded onto 4–12% gradient gels, Bolt Bis Tris (Thermo), before they were transferred to BioTrace™ NT Nitrocellulose membrane (Pall Corporation, Port Washington, NY) in pre-cooled 1X Transfer Buffer (25 mM Tris, 190 mM glycine, 20% methanol, pH 8.5). The membranes were washed 3 times with TBS-T (10 × 1.5 M NaCl, 0.5 M Tris, pH 7.6, 0.1% Tween 20), blocked with 1% BSA for 1 h, and incubated overnight with primary antibodies diluted 1:1000 in 1% BSA at 4 °C. After washing, membranes were incubated with an appropriate IgG secondary antibody for Western analysis, diluted 1:10,000 in 1% BSA in TBS-T. Proteins were detected using a LI-COR Odyssey 9120. ImageJ was used for quantification.

### Immunoprecipitation

For immunoprecipitation, a 200 µL input sample was collected, and the remaining lysate volume was pre-cleared with SureBeads™ Protein G Magnetic Beads (Bio-Rad, Hercules, CA, USA) for 1 h at 4 °C. The beads were removed and discarded. The supernatant was then incubated with fresh beads and 1 μg antibody overnight at 4 °C. After washing the beads 4 times in Lysis Buffer, they were resuspended in 20 µL SDS-Loading Dye (4X SDS-Loading Dye is 0.25 M Tris-HCl, pH 6.8, 40% glycerol, 8% SD, 0.01% Bromophenol Blue) and denatured by heating to 95 °C for 10 minutes before Western analysis.

### siRNA transfection for Ubc9 knockdown

Two Ubc9-specific siRNA sequences (SASI_Hs01_00124119 and SASI_Hs01_00124120) and a non-targeting siRNA control were purchased from Sigma Aldrich and diluted in RNAse-free water to 20 μM. Cells were seeded at 40–50% confluency in 6-well plates and allowed to adhere for 24 h. For transfection, siRNAs were diluted to 20 nM in Opti-MEM® (Thermo) with 4 μL of Lipofectamine® 3000 (Thermo) to a final volume of 200 μL. After incubating at room temperature for 30 min, the transfection complexes were added to cells in fresh Opti-MEM® and incubated at 37 °C, 5% CO_2_. After 5 h, the medium was replaced and cells incubated for 24–48 h before protein lysates were created for Western analysis.

### TAK981 and MG132 treatment

Cells were seeded at 40-50% confluency in 6-well plates and allowed to adhere for 24 h. For TAK981 (10 μM, Selleck), cells were treated for 48 h before protein isolation. For MG132 (20 μM, Selleck), cells were seeded at 5 × 10⁵ for 24 h and treated for 6 h the following day. DMSO at 0.1% was used as the vehicle for all treatments.

### Transient transfection

Lipofectamine 2000 (Thermo) was used to transiently transfect MCF10A cells that had been seeded in a 6-well dish for 24 h prior with wild-type EphB4, vector only, EphB4 K616R and Lipofectamine only (Mock). Cells were lysed and protein lysates made after 48 h.

### Quantitative real-time PCR

RNA from cells over-expressing EphB4, K616R and the Vector Only (Vo) were extracted using Trizol (Thermo). Quantitative Real Time PCR (RT-PCR) was then performed as previously described [25]. Briefly, extracted RNA was reverse transcribed using Superscript III reverse transcriptase (Thermo). *EPHB4* quantitative real-time PCR was performed using a SYBR green PCR master mix (Thermo) with *HMBS* amplified as the reference gene. Reactions were performed using the ViiA 7 Real-Time PCR Machine (Thermo).

### Wound closure assay

Cells were seeded into 96-well ImageLock plates (Essen BioScience, MI, USA) and allowed to adhere overnight. Uniform scratches were then generated in each well using a WoundMaker^TM^ (Essen BioScience). After scratching, the medium containing detached cells was removed and replaced with 200 μL of fresh medium. The plate was then placed into the IncuCyte SX5 for live-cell imaging. For each derivative cell line, six replicate wells were analysed, with two images captured per well (12 images total per line) every 2 h for 48 h. Data was analysed using the IncuCyte ZOOM software.

### Statistics

Statistical analyses were performed using GraphPad Prism v6.01 (GraphPad Software, Boston, MA). Statistical significance was determined by one-way ANOVA with multiple comparisons.

## Results

### EphB4 is SUMOylated in prostate cancer cell lines

**Fig. 1:**
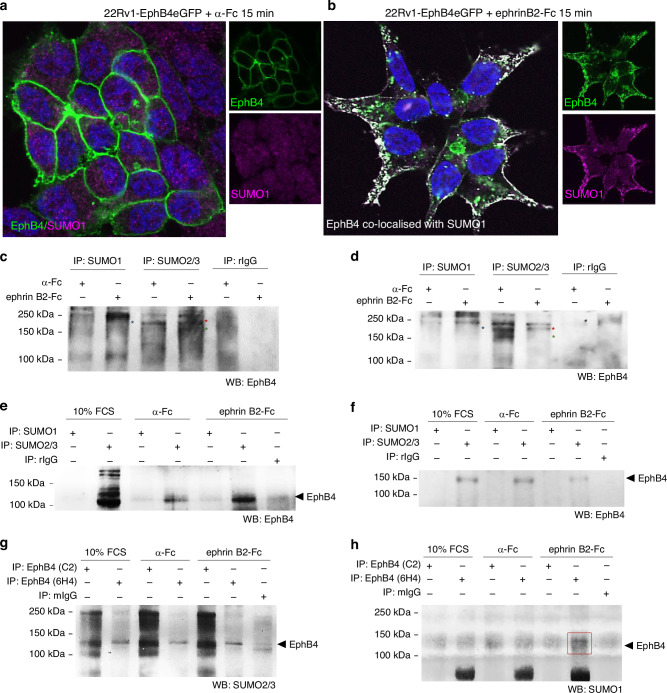
EphB4 is SUMOylated in prostate cancer cells. **a**, **b** Confocal microscopy showing co-localisation of EphB4-eGFP (green) overexpressed in 22Rv1 cells with SUMO1 (magenta). Cells were treated with **a**. clustered Fc protein only (α-Fc) or **b**. Stimulated with soluble clustered ligand (ephrinB2-Fc) for 15 min. EphB4-eGFP and SUMO1 (magneta) co-localisation is shown in white. Nuclei are counterstained with DAPI (blue). 22Rv1 (**c**) or LNCAP (**d**) cells were serum starved for 2 h and then stimulated for 15 min with clustered ephrin B2-Fc or Fc control (α-Fc). Total cell lysates were immunoprecipitated using anti-SUMO1, anti-SUMO2/3 or control rabbit IgG (rIgG) followed by Western analysis with an EphB4 antibody. SUMO1-ylated EphB4 is indicated by blue asterisks and SUMO2/3-ylated EphB4 is indicated by the red and green asterisks. PC3 cells (**e**) or DU145 cells (**f**) were cultured in 10% FCS or serum starved for 2 h before stimulation for 15 min with ephrin B2-Fc or Fc control. For both cell line samples, immunoprecipitation was performed using anti-SUMO1, anti-SUMO2/3 or rIgG (negative control) followed by EphB4 Western analysis. **g**, **h** PC3 cells were cultured in 10% FCS or serum starved for 2 h before stimulation for 15 min with ephrin B2-Fc or Fc control. Immunoprecipitation was performed using two anti-EphB4 antibodies (C2 and 6H4) [25], with the sample divided into half for Western analysis using either SUMO2/3 (**g**) or SUMO1 (**h**) antibodies. Mouse IgG was used as a negative control. These experiments were repeated at least three times with similar results obtained.

Conjugation and deconjugation of SUMO to target proteins is highly dynamic. Given that four other RTKs (EGFR, IGF-IR, PGDFRα and ErbB4) are all reported to be SUMOylated upon ligand stimulation, we over-expressed EphB4 with a C-terminal eGFP tag (EphB4-eGFP) in the prostate cancer cell line 22Rv1, then serum starved the 22Rv1 EphB4-eGFP cells for 16 h, prior to stimulation with the control clustered Fc protein only (α-Fc) or clustered soluble ligand (ephrinB2-Fc) for 15 min (Fig. 1a, b). Localisation of EphB4-eGFP and SUMO1 was first determined by immunofluorescence. In the unstimulated control cells (α-Fc), EphB4-eGFP remained on the cell membrane (Fig. 1a, green fluorescence) and SUMO1 was seen through-out the cytoplasm and nucleus (Fig. 1a, magenta fluorescence). Ligand stimulation resulted in significant co-localisation of EphB4-eGFP with SUMO1 at the cell membrane and in the cytoplasm (Fig. 1b, white signal in overlay). This co-localisation was not seen in the 22Rv1 cells transfected with the empty vector and expressing eGFP only (Vo-eGFP) even upon stimulation (Supplementary Fig. 1A and 1B). A second cell line model using MCF10A cells where EphB4 was overexpressed and the cells stimulated, gave the same result (Supplementary Fig. 1C and 1D).

To determine if EphB4 is directly modified with SUMO1 and/or SUMO2/3 following ligand stimulation and not simply moved into a complex with other proteins that are also SUMOylated, parental 22Rv1 cells were then stimulated as above, and total protein lysates were used for immunoprecipitation (IP) of SUMO1 or SUMO2/3. Western analysis of the immunoprecipitated proteins using an EphB4-specific antibody identified bands that appeared enriched in the samples from ephrin-B2 stimulated cells (Fig. 1c). Consistent with the IF result, a strong band was detected in the SUMO1 IP from cells treated with ephrin-B2, when compared with the control α-Fc only sample (Fig. 1c, blue asterisk). A similar band was also seen in the SUMO2/3 IP and was also increased in the cells treated with ephrin-B2 ligand (Fig. 1c, red asterisk). A lower molecular weight band suggests that some EphB4 in these cells is already modified by SUMO2/3 and this level is not altered by ligand stimulation (Fig. 1c, green asterisk).

To determine if EphB4 SUMOylation occurs in other prostate cancer cells, a similar experiment was then performed using the LNCaP prostate cancer cell line. A band of a similar size to that identified in the 22Rv1 cells was also detected in the LNCaP cell samples (Fig. 1d, blue asterisk). In these cells, EphB4 modified with SUMO2/3 was again present prior to ligand stimulation but appeared to be reduced by ligand stimulation (Fig. 1d, red and green asterisks).

Collectively, these results suggest that EphB4 is modified with SUMO2/3 during conditions of normal cell culture and is then modified with SUMO1 after ligand stimulation. Evidence from many studies of various substrate proteins has shown that modification with SUMO1 and SUMO2/3 causes distinct functional outcomes and a SUMOylation switch may regulate EphB4 [27]. To test this, EphB4 SUMOylation was examined in the androgen-independent prostate cancer cell line PC3 grown in medium containing 10% serum and compared with cells that had been serum starved (Fig. 1e). PC3 was chosen for this experiment because EphB4 produced by PC3 cells is mostly localised in the cytoplasm where it is predicted to function in a ligand-independent manner [10, 26]. Accordingly, EphB4 in PC3 cells does not appear to be modified with SUMO1 even when a ligand is added. Several EphB4 immunoreactive bands were identified in the SUMO2/3 IP sample from cells grown in full serum medium, but were not strongly detected in the sample from cells that were serum starved. This suggests that EphB4 in PC3 cells grown under normal conditions is modified with SUMO2/3. EphB4 SUMO2/3 modification is lost during the 16 h serum starvation and as expected, modification with SUMO2/3 was not increased by the addition of ligand, highlighting that EphB4 is modified in response to a separate growth signal stimulus. A similar result was also seen for DU145 cells, which also have cytoplasmic EphB4 (Fig. 1f) [10, 26].

Finally, to confirm that EphB4 in PC3 cells is modified with SUMO2/3 under normal growth conditions (medium with 10% serum), an immunoprecipitation was performed using EphB4 antibodies with subsequent Western analysis using either an anti-SUMO2/3 antibody or an anti-SUMO1 (Fig. 1g, h). The SUMO2/3 antibody precipitated an EphB4 reactive band at the predicted size of SUMO-modified EphB4 but this band was not detected in the SUMO1 pulldowns (Fig. 1h). Two different EphB4 antibodies, C2 and 6H4 [26], precipitated a SUMO2/3 band of approximately 140 kDa which is consistent with SUMO-modified EphB4. The C2 antibody also immunoprecipitated another higher molecular weight EphB4 band (250 kDa) that was also detected using the SUMO2/3 antibody, but not detected with the SUMO1 antibody and is possibly a result of polySUMOylation.

### *UBE2I* siRNA knockdown correlates with a loss of EphB4

**Fig. 2:**
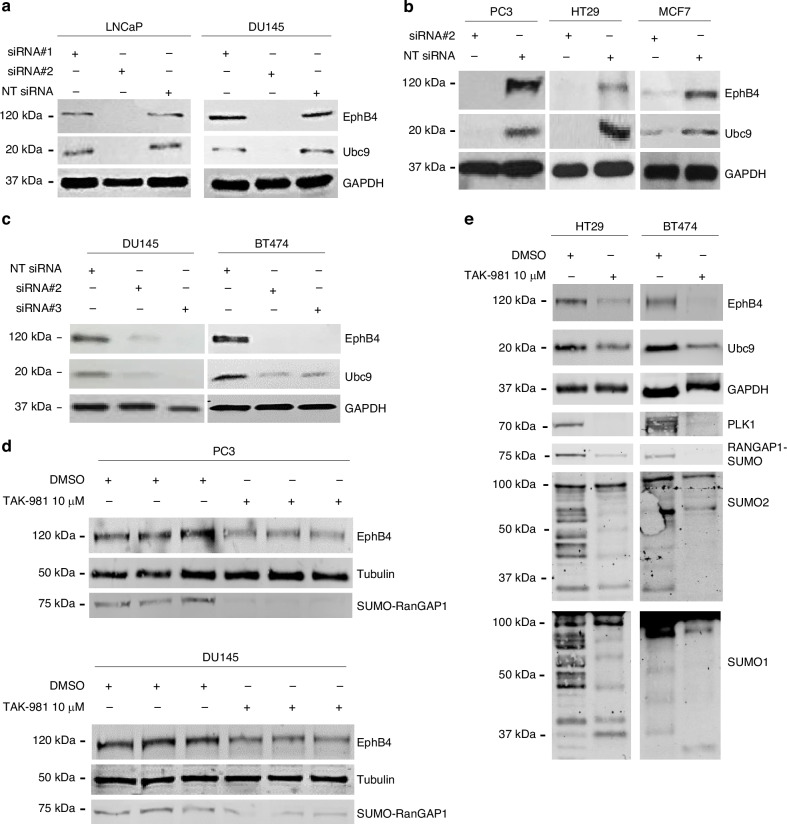
EphB4 protein levels are reduced following *UBE2I* knockdown. **a** LNCaP and DU145 cells were transfected with two siRNA sequences targeting UBE2I (siRNA #1 and #2) or a scrambled non-targeted siRNA sequence (control siRNA) followed by Western analysis. **b** *UBE2I* siRNA#2 was used PC3, HT29 and MCF7 cells and Western analysis was conducted as in (**a**). **c** A third siRNA sequence (siRNA#3) targeting *UBE2I* was tested in DU145 and BT474 cells and knockdown was confirmed as in (**a**). These experiments were repeated at least three times with similar results obtained. **d** Western analysis of total protein lysates from HT29 and BT474 cells treated with TAK-981 (10 µM, 48 h) and Western analysis using antibodies specific to SUMO1, SUMO2/3, Ubc9, EphB4, PLK1 and RanGAP1. **e** PC3 and DU145 cells treated with TAK-981 (10 µM, 48 h) analysed as in (**a**) with α-Tubulin and RanGAP1 included as additional controls. These experiments were repeated twice with similar results obtained.

Ubc9 is the sole E2 conjugating enzyme for SUMOylation encoded by the *UBE2I* gene in the human genome [13, 14]. Knockdown of *UBE2I* or inhibition of Ubc9 can identify proteins that require SUMOylation for stability, cellular localisation and function [27, 28]. An siRNA approach was used to knock down *UBE2I* in DU145 and LNCaP cells. Initially, two MISSION® siRNA sequences were tested, siRNA#1 (SASI_Hs02_00335499, sequence start 666) and siRNA#2 (SASI_Hs01_00124119, sequence start 961), with the MISSION^®^ siRNA Universal Negative Control #1 used as the non-targeting siRNA negative control (NT siRNA). Complete loss of Ubc9 was seen in both cell lines with siRNA#2 and this correlated with complete loss of EphB4 protein (Fig. 2a). Knockdown of both Ubc9 and EphB4 with siRNA#2 was confirmed using three more cancer cell lines, PC3, colon cancer line HT29 and breast cancer line MCF7 (Fig. 2b). A third siRNA sequence was then tested (SASI_Hs01_00124120, sequence start 478) and this sequence showed Ubc9 knockdown efficiency comparable to siRNA#2 and also resulted in a loss of EphB4 (Fig. 2c).

### SUMO inhibition with small molecules also correlates with a loss of EphB4

To confirm that SUMOylation influences EphB4 levels, a second approach to modulating SUMOylation was used. TAK-981 (Takeda) is a general inhibitor of the E1 SUMO-activating heterodimer enzyme (SAE1-SAE2) and functions by forming an adduct with the SUMO E1 enzyme, preventing transfer of the activated SUMO to Ubc9 and ultimately preventing modification of substrate proteins [29]. TAK-981 (subasumstat) is currently being clinically assessed in Phase 1b/2 trials against cancer [30, 31]. PC3 and DU145 cells were then treated with 10 µM TAK-981 for 48 h and EphB4 was lost from both cell lines (Fig. 2d). A loss of highly SUMOylated RanGAP1 [32, 33] confirms the TAK-981 was preventing SUMOylation as expected (Fig. 2d). To confirm SUMOylation of EphB4 is required to cancer cells in general, HT29 colon cancer cells and BT474 breast cancer cells were treated for 48 h with 10 µM TAK-981. Western analysis confirmed general knockdown of SUMOylation with less SUMO1 and SUMO2/3 modified proteins detected in the treated lines when compared with vehicle (DMSO) controls (Fig. 2e). A loss of SUMOylated RanGAP1 [32, 33] and PLK1 [34] confirms that these proteins could be used as markers for general SUMOylation inhibition (Fig. 2e). In the treated cells there was less EphB4 and Ubc9 detected indicating that an active SUMOylation cascade is required for EphB4 and Ubc9 stability (Ubc9 itself can be SUMOylated) (Fig. 2e). Together, these results show that SUMOylation is required for EphB4 stability in cancer cell lines.

### K616 controls EphB4 stability and is a SUMOylation site

**Fig. 3:**
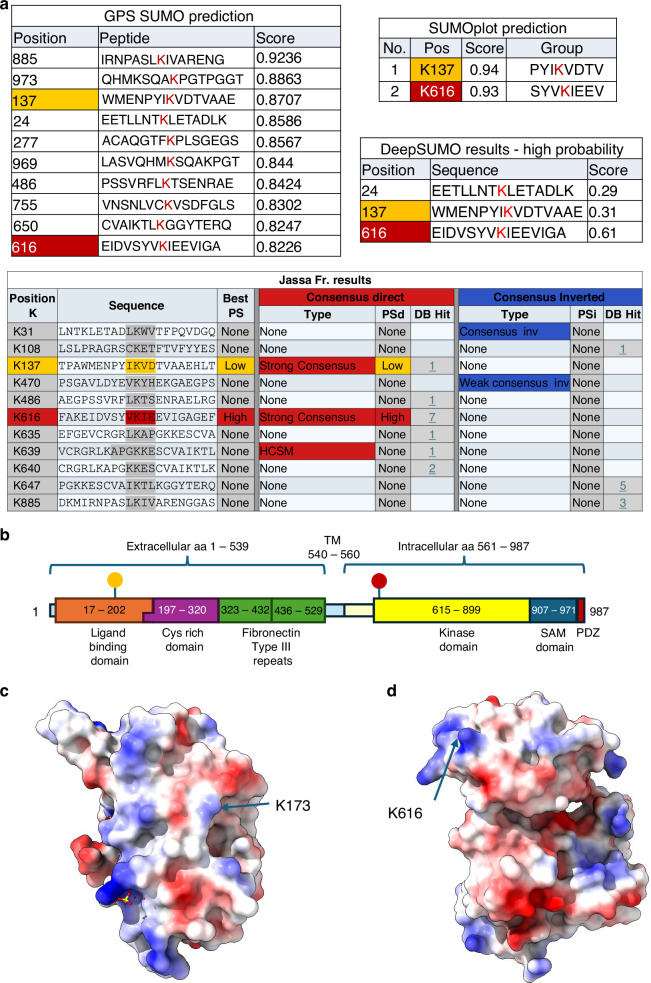
In silico prediction of EphB4 SUMOylation sites and motifs. **a** Output tables from four different in silico SUMOylation prediction tools (GPS SUMO, SUMOplot, DeepSUMO and Jassa.Fr.), highlighting K137 and K616 as high probability consensus sites. **b** Schematic of EphB4 domains showing K137 (orange indicator) in the extracellular ligand binding domain and K616 (red) in the intracellular kinase domain of the EphB4 protein. Domains: Ligand Binding domain (orange), Cysteine-rich Sushi/EGF domain (purple), Fibronectin Type III repeats (green); Transmembrane domain (TM, light blue), juxtamembrane sequence (light yellow), Kinase domain (yellow), SAM domain (dark teal) and PDZ motif (red). Amino acid residues for each domain are indicated. ChimeraX modelling of the EphB4 domains showing surface exposure of K137 in the ligand binding domain (**c**, PDB: 2BBA) and K616 in the kinase domain (**d**, PDB: 6FNK).

SUMOylation often occurs on a lysine within a consensus sequence ψKxD/E where ψ refers to a large hydrophobic amino acid, K is the target lysine, x is any amino acid, and D/E is an aspartic acid or glutamic acid) [35–37]. While consensus motifs facilitate SUMOylation, they are not strictly required and SUMO can modify lysines outside these motifs [37]. To determine if EphB4 has a high probability SUMO motif, the amino acid sequence (Uniprot P54760) was analysed using available online SUMO motif prediction programs SUMOPlot™ (https://www.abcepta.com/sumoplot), GPS SUMO (https://sumo.biocuckoo.cn/) [38], Jassa Fr. (http://www.jassa.fr/) [39] and Deep SUMO (http://deepsumo.renlab.org/ server.html) [40]. All four programs identified two high probability motifs at K137 (IKVD) and K616 (VKIE, Fig. 3a). K137 is within the extracellular ligand binding domain and K616 within the N-lobe of the kinase domain (Fig. 3b). In the crystal structure of EphB4, both residues are on the surface of the protein and accessible for potential SUMOylation (Fig. 3c, d) but K616 was considered the most likely of these to be modified given this part of the protein is intracellular and directly accessible to the cytoplasmic SUMOylation proteins and enzymes.

**Fig. 4:**
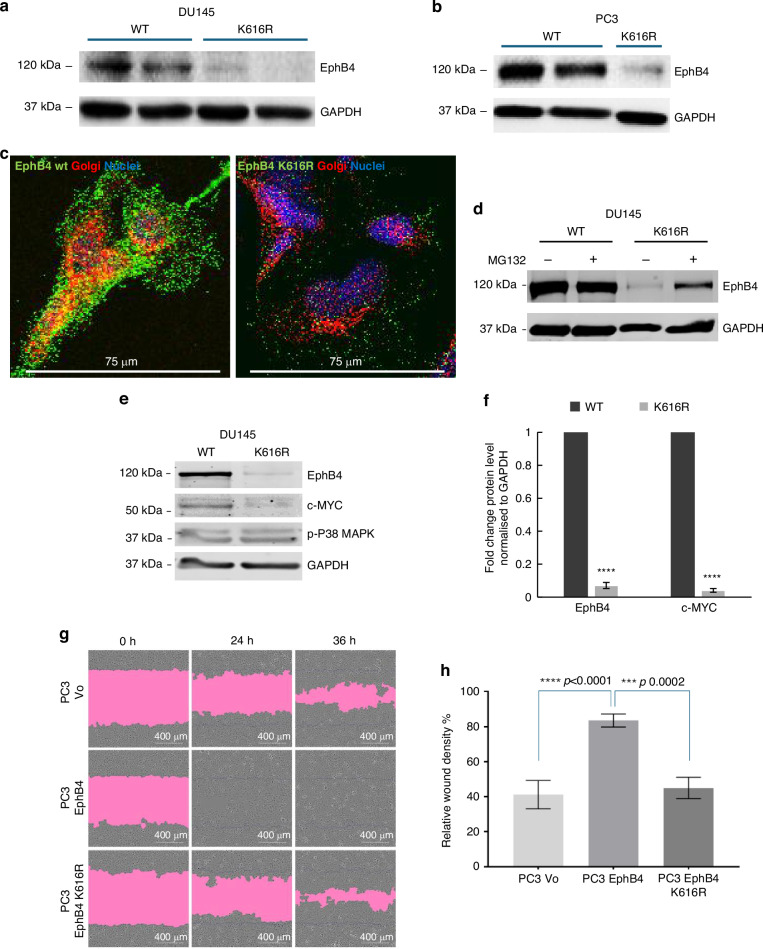
SUMOylation at K616 stabilises EphB4. DU145 (**a**) and PC3 (**b**) cells were transfected to create stable cell lines over expressing EphB4 (WT) or EphB4-K616R (K616R). Western analysis was conducted using an EphB4-specific antibody and GAPDH is shown as a loading control. This experiment was repeated three times with similar results obtained. **c** Immunofluorescence of EphB4 (green) and GM130 Golgi marker (red) in DU145 cells expressing WT (Panel 1) or K616R (Panel 2). Nuclei were stained with DAPI (blue). **d** Western analysis detecting EphB4 proteins in DU145 derivative cells expressing either wild type or mutant EphB4 K616R after 6 h of treatment with either DMSO vehicle (−) or 20 μM MG132 (+). GAPDH was used as the loading control. *n* = 3. **e** Western analysis detecting level of EphB4, MYC and p-P38 MAPK proteins in DU145 derivative cells expressing either wild type or K616R mutant EphB4. GAPDH was used as the loading control. *n* = 3. **f** Densitometry data for the EphB4 and c-MYC expression are presented as mean ± standard deviation from three different independent experiments (****P < 0.0001). **g** Scratch wound healing assay comparing PC3 derivative lines stably over-expressing EphB4 WT, EphB4 K616R or the empty vector (Vo) over a total of 36 h. Data was collected using an IncuCyte SX5 (Sartorius) and analysed using the IncuCyte ZOOM software. In each image (10X magnification), the original edge of the wound at 0 h is shown in blue, with the cell-free area at different time points indicated in pink. **h** Quantification of relative wound density (%) at 24 h comparing PC3 derivative lines with stable expression of EphB4 WT, EphB4 K616R or the empty vector (Vo). Statistical analysis using one-way ANOVA was performed with GraphPad Prism using data collected from two independent experiments using 6 wells per cell line and 2 data points collected per well (n = 24).

A site-directed mutagenesis approach was used to substitute lysine 616 with an arginine (K616R), preserving the positive charge but preventing SUMOylation at this site [34, 37]. Wildtype and mutant EphB4 K616R proteins were then over-expressed in the two prostate cancer cell lines, DU145 and PC3 (Fig. 4a, b, respectively). In both cell lines, a band consistent with the mutant protein was not readily detected by Western analysis despite confirmed expression of EphB4 mRNA in the derivative cell populations (Supplementary Fig. 2a, b). A similar lack of detectable EphB4 K616R protein was observed following transient transfection in MCF10A cells (Supplementary Fig. 2c). Consistent with these findings, immunofluorescence analysis showed strong EphB4 signal in DU145 cells expressing the wild-type protein, but minimal signal in those expressing the K616R mutant (Fig. 4c, panel 1 vs. panel 2). Given that SUMOylation is known to enhance protein stability, we investigated whether the EphB4 K616R protein was being translated but rapidly degraded via the proteasome. DU145 cells expressing either wild-type or mutant EphB4 K616R proteins were treated with the proteasome inhibitor MG132 (20 µM for 6 h). In 4 repeats of this experiment, an improvement in the amount of EphB4K616R was seen, confirming that the exogenous mutant protein EphB4K616R is degraded under normal conditions (Fig. 4D).

### K616 on EphB4 promotes MYC signalling and supports migration

Over-expression of the oncogenic transcription factor *c-myc* is frequently observed in prostate cancer and drives tumour initiation. The expression of EphB4 and *c-myc* has been positively correlated by several studies [8, 9] in LNCaP, PC-3 and 22Rv1 prostate cancer cell lines. Western analysis was used to determine if the level of c-MYC is influenced by the over-expression of wild-type EphB4 or EphB4 K616R in DU145 cells. More c-MYC was detected in the wildtype expressing cells than in the EphB4 K616R cell,s suggesting that *c-myc* expression is reduced when EphB4 cannot be SUMOylated at K616 (Fig. 4E, F). In this experiment using DU145 cells, phosphorylation of p38 MAPK (Thr 180/Tyr182) was not altered even though it has been reported to correlate with EphB4 knockdown in PC3 cells [8].

Previous published experiments have shown that EphB4 contributes to metastatic potential by increasing migratory ability [6, 7, 10, 26]. For this reason, a wound closure migration experiment was used to compare PC3 cells over-expressing empty vector (PC3-Vo), wildtype EphB4 (PC3-EphB4) or EphB4 K616R (PC3-EphB4 K616R). As expected, PC3 cells over-expressing EphB4 exhibited greater migratory capacity and closed the wound faster than the empty vector cells (*P* < 0.001). In contrast, the EphB4 K616R cells showed a statistically significant reduction in migratory capacity when compared to the wildtype EphB4 cells, reaching levels comparable to the empty vector control (Fig. 4g, h). This suggests that the enhanced migration conferred by wild-type EphB4 is dependent on SUMOylation at lysine 616, and this modification is essential for EphB4’s promigratory function.

### K616 is the major, but not only, SUMOylation site on EphB4

**Fig. 5:**
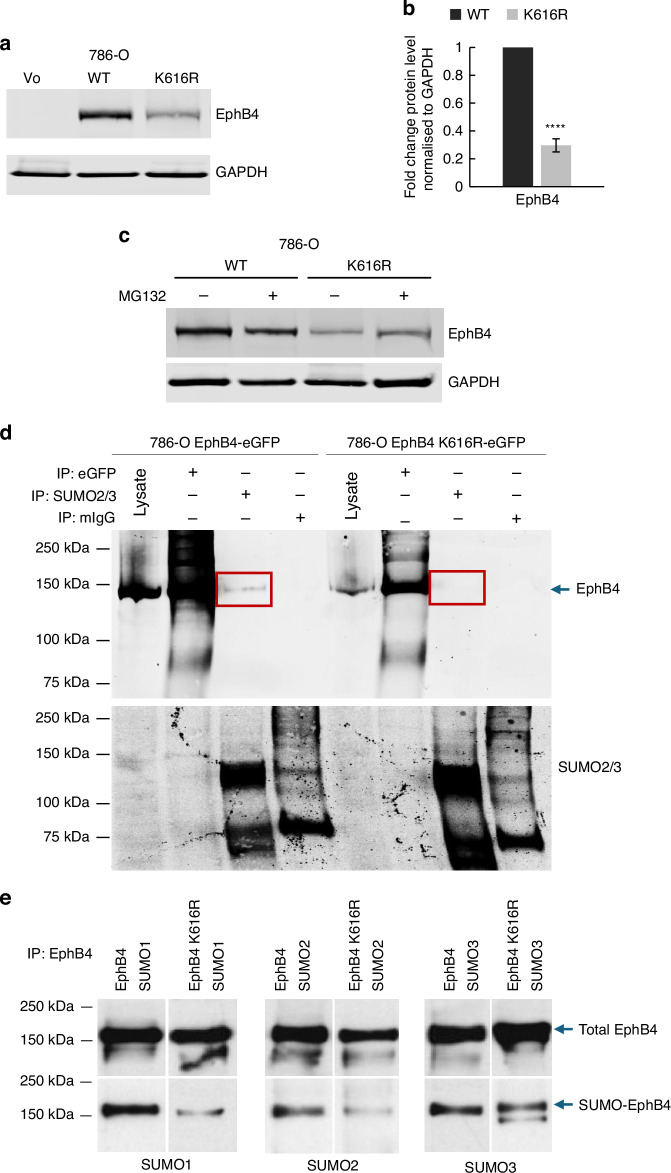
K616 the major, but not only, site for EphB4 SUMOylation. 786-O cells over-expressing eGFP-tagged EphB4 or EphB4 K616R were created. **a** Western analysis confirming expression of EphB4-eGFP or EphB4 K616R-eGFP in the derivative cells. **b** Densitometry was used to quantify the WT and K616R protein levels, with K616R determined to be 30 ± 5% of the WT protein level. *n* = 3. ****P < 0.0001. **c** Western analysis detecting EphB4 proteins in 768-O derivative cells expressing either wild type or mutant EphB4 K616R after 6 h of treatment with either DMSO vehicle (−) or 20 μM MG132 (+). GAPDH was used as the loading control. *n* = 4. **d** Protein lysates were made and immunoprecipitation was conducted using an eGFP-specific antibody (to immunoprecipitate the EphB4-eGFP proteins) or a SUMO2/3-specific antibody. Duplicate blots were made and probed with an anti-EphB4 antibody (top panel) or a SUMO2/3 antibody (bottom panel). SUMO2/3-modified EphB4 proteins are indicated in the red boxes. *n* = 3. **e** 786-O cells over-expressing both EphB4 wildtype or EphB4 K616R with mScarlet-SUMO1, mScarlet-SUMO2 or mScarlet-SUMO3 were created. Protein lysates were made and immunoprecipitation was conducted using an EphB4-specific antibody. Samples were divided equally to make two duplicate blots for Western analysis using an EphB4-specific antibody (top panel) and a SUMO-specific antibody (SUMO1 – Abcepta; SUMO2/3 – Novus) (bottom panel).

To confirm the absence of mutant EphB4 K616R protein was due to its proteasomal degradation and not a consequence of normal endogenous EphB4 signalling and recycling functions, EphB4-eGFP and K616R-eGFP proteins were then expressed in the 786-O kidney cancer cell line, which lacks endogenous EphB4 protein (Fig. 5a). WT EphB4-eGFP was expressed well by the 786-O cells but as previously observed, the EphB4 K616R-eGFP protein was detected at a lower level (Fig. 5b). Levels of the mutant EphB4 K616R-eGFP protein were consistently increased (2–5 fold) upon MG132 treatment, indicating that the mutant EphB4 K616R-eGFP protein is unstable and targeted for proteasomal degradation, consistent with a stabilising role for SUMOylation at lysine 616 (Fig. 5c).

To determine if EphB4 is SUMOylated only at lysine 616 (K616), whole-cell lysates were made from 786-O cells expressing either the wild-type EphB4-eGFP or the K616R-eGFP mutant proteins. A sample of each lysate was reserved for a control before the rest was divided into three separate aliquots each. EphB4 was immunoprecipitated from the first aliquot using a validated anti-eGFP antibody (Novus). Proteins modified with SUMO2/3 were immunoprecipitated from the second aliquot using the validated mouse monoclonal anti-SUMO2/3 (Novus). The third sample was incubated with an irrelevant mouse IgG preparation and used as a negative control. The immunoprecipitated material was then divided into two samples and resolved by SDS-PAGE to generate duplicate Western blots. The first blot was probed with an anti-EphB4 antibody (Cell Signalling, 14960), confirming successful immunoprecipitation of both EphB4-eGFP and EphB4-K616R-eGFP proteins, visible as strong bands in the relevant lanes and consistent with the band detected in the lysate samples (Fig. 5d). The second duplicate blot was probed with an anti-SUMO2/3 antibody to detect the SUMOylated fraction and immunoprecipitation of SUMOylated proteins was successful. SUMOylated EphB4-eGFP was detected as a faint band when the sample immunoprecipitated with the anti-SUMO2/3 antibody was probed with the EphB4 antibody. This band could not be detected in the lysate from the 786-O cells expressing EphB4-K616R-eGFP. These findings confirm that the major site for SUMOylation conferring stability to EphB4 is K616.

Finally, to determine if EphB4 is SUMOylated only at lysine 616 (K616), wild-type EphB4 or the K616R mutant was co-expressed in 786-O cells with SUMO1, SUMO2, or SUMO3, each tagged with mScarlet. EphB4-SUMO conjugates were immunoprecipitated from whole-cell lysates using a rabbit monoclonal anti-EphB4 antibody (Cell Signalling, #14960). The immunoprecipitated material was divided into two samples and resolved by SDS-PAGE to generate duplicate Western blots (Fig. 5e). The first blot was probed with a mouse monoclonal EphB4 antibody (Zymed, clone 3E7G9), confirming successful immunoprecipitation of both wild-type and K616R EphB4 proteins, visible as a strong band in each sample (Fig. 5e – top panel). The second blot was probed with a rabbit RFP antibody (Thermo) and a band of the same size was detected, identifying this as the SUMOylated EphB4 in the sample (Fig. 5e – bottom panel). In each of the cell line samples from 786-O expressing the EphB4 K616R protein, the intensity of the SUMOylated band was less but still present, showing that EphB4 can be SUMOylated on residues in addition to K616.

## Discussion

Here we demonstrate that SUMOylation is a post-translational modification that regulates EphB4 protein stability. We establish that EphB4 is modified by both SUMO1 and SUMO2/3. SUMO1 conjugation occurs rapidly, within 15 minutes following stimulation with clustered ephrinB2, consistent with previous reports for other receptor tyrosine kinases (RTKs), where ligand binding triggers SUMO1 modification [16–18]. In contrast, under basal conditions, EphB4 is constitutively SUMOylated by SUMO2/3, and this pattern is not altered by ligand stimulation. This may indicate the presence of distinct SUMOylation sites on EphB4, one responsive to ligand and SUMO1, and another constitutively modified by SUMO2/3. Alternatively, a single lysine residue could undergo SUMO “switching” [41, 42], or a SUMO2/3 chain may be capped by SUMO1 [43].

SUMOylation typically modifies only a small fraction of a protein at any given time (often <1–5%), yet its impact can be magnified through rapid conjugation–deconjugation cycles (modification and removal can occur within seconds to minutes) [13, 44]. There are also paralogue-specific effects reported, with modification by SUMO1 versus SUMO2/3 at the same lysine producing distinct outcomes, including altered stability, localisation, or recruitment of SIM-containing protein binding partners, effectively acting as a regulatory switch [32, 44–47]. Transient and/or low-abundance SUMOylation can initiate durable downstream events, which explains why preventing modification at a single site can have profound effects on cell behaviour and viability. This fundamental feature of SUMO signalling has been called the SUMO enigma [35].

The contribution of the ligand-induced and constitutive modifications to EphB4 functions remains to be determined. Like other RTKs that are modified with SUMO1, modification of EphB4 with SUMO1 in response to ligand stimulation might be required for activation of specific downstream signalling pathways, might cause a change in localisation (into the nucleus) or might alter the conformation of EphB4 to provide new protein-protein interaction interfaces [15–24]. Our data suggests EphB4 is modified with SUMO1 on residues other than K616 and future studies will explore this further – identifying the modified residues and exploring the contribution to signalling, cellular localisation and stability. Here we have shown that EphB4 is modified with SUMO2/3 on K616 and this increases EphB4 stability. This could be achieved in a number of ways, including 1) blocking ubiquitin-mediated degradation, (2) competing with other destabilising post-translational modifications (e.g., acetylation), (3) recruiting stabilising SIM-containing proteins, (4) changing the subcellular localisation and in some cases, (5) stabilising protein conformation [13, 14, 32].

SUMOylation is increasingly reported as a key regulatory mechanism for RTKs, affecting cellular localisation, protein-to-protein interactions, and stability. Extensive research has been conducted on IGF-1R SUMOylation. Upon ligand stimulation, IGF-1R is phosphorylated, then SUMO1-ylated, facilitating nuclear translocation where it functions as a transcriptional co-activator of the LEF1/TCF complex, promoting expression of genes such as *cyclin D1*, *axin2*, and *SNAI2* [17, 48]. Mutation of three evolutionarily conserved lysine residues (K1025, K1100 and K1120) to arginine prevents nuclear localisation [17]. This IGF-1R triple SUMO mutant (TSM) protein is also less stable than wildtype protein, with 59% lost in the 24 h after treatment with cycloheximide compared with loss of only 15% of the wild-type receptor [17]. Similarly, knockdown of the E3 ligase, RanBP2, decreases IGF-1R stability, supporting the role of SUMOylation as protective of IGF-1R from degradation [17]. Packham et al. (2015a, b) suggested that adequately SUMOylated IGF-1R is targeted to the nucleus and non-SUMOylated protein is degraded [16, 18]. Titone et al. (2018) report that under conditions of serum starvation, IGF-1R accumulates in the nuclei of normal corneal epithelial cells independent of IGF-1 in a stress response requiring modification with SUMO2/3 and an interaction with IGFBP-3 [49]. This serves to maintain levels of IGF-1R in the nucleus and support cell survival.

Another Eph family member, EphB1, has also been shown to undergo SUMOylation [15]. Mutation of K785 significantly reduced SUMO1 conjugation and was associated with increased tumour growth in xenografts, suggesting that SUMOylation at this residue exerts a tumour-suppressive effect. SUMOylation at K785 also reduced downstream PKCγ phosphorylation, a key signalling event, supporting the idea that this modification serves as a regulatory “off switch” for ligand-dependent signalling. Notably, K785 lies within the kinase domain and is conserved in 12 of the 14 Eph receptors, excluding the kinase-dead EphA10 and EphB6. This conservation suggests a potential evolutionary link between SUMOylation and Eph kinase function. In line with this, we show that EphB4 is SUMO1-modified following ephrinB2 stimulation. Although the functional consequences of this event require further investigation, prior studies have reported tumour-suppressive effects of ligand-activated EphB4 signalling [10], hinting at a possible parallel mechanism.

In our study, we used a site-directed mutagenesis approach to show that K616 is SUMOylated for the stability of EphB4 using SUMO2/3. Chen et al. (2018) included EphB1 K620, a lysine in a position consistent with EphB4 K616, in their mutagenesis study but observed no change to SUMOylation with SUMO1 or EphB1 stability [15]. There are several possible explanations for this discrepancy. Chen et al. (2018) used HEK293 as their model cell line, which, as already stated, expresses ephrinB1 and may promote ligand-dependent EphB1 signalling through cell-to-cell contact [15]. By contrast, our experiments were conducted using prostate cancer cell lines DU145 and PC3, where the cognate ligand for EphB4, ephrinB2, is poorly expressed and EphB4 shows limited plasma membrane presentation [10, 26]. Secondly, the conjugation step of SUMOylation is performed by Ubc9, the sole enzyme encoded by the human genome, but substrate specificity and efficiency are influenced by various E3 and E4 ligases with tissue- and compartment-specific expression and ligase expression may also differ between these cell lines [50, 51]. Finally, this difference may reflect an intrinsic difference between EphB1 and EphB4, two closely related but functionally different proteins.

Although the mutation of EphB4 at K616 significantly reduced its SUMOylation, it did not completely abolish the modification at this lysine. Several explanations could account for this observation. First, EphB4 likely contains multiple SUMOylation sites, as is reported for other RTKs [17–19] and additional lysine residues may contribute to its overall SUMOylation status in a cancer cell line. This has been reported for IGF-1R, where simultaneous mutation of three conserved lysine residues was required to fully disrupt SUMOylation and observe the functional consequences [17–19]. In silico predictions suggest that K137 may be a stronger candidate for SUMOylation than K616, making it a logical next target for mutagenesis studies. EphB4 also has a lysine (K781) in the same position as SUMOylated K784 of EphB1. Second, it is possible that a compensatory mechanism allows SUMOylation to occur at alternative lysine residues, albeit less efficiently, when K616 is no longer available. Taken together, our data strongly support the role of K616 in regulating EphB4 protein stability and, by extension, its functional role in prostate cancer cells. However, K616 is not the sole site of SUMOylation on EphB4, and further investigation is warranted to fully map the SUMOylation landscape and its implications for EphB4 signalling and stability.

EphB4 has been reported to be over-expressed in 66% of prostate cancer tissue and our study establishes that SUMOylation confers stability to EphB4 in prostate cancer cell lines, offering a potential mechanism for this over-expression in tumours. Eph receptors have both ligand-dependent and ligand-independent functions as well as kinase-dependent and kinase-independent functions [10, 52]. The growing collection of data linking SUMOylation to receptor tyrosine kinases adds a further dimension of complexity to our understanding of Eph receptor signalling. A better understanding of how SUMOylation is regulated and how it intersects with other signalling pathways may help reconcile the dual tumour promoting and tumour-suppressive roles attributed to Eph receptors, even within the same tissue context.

## Supplementary information

Supplementary Fig. Legends

Supplementary Fig. 1

Supplementary Fig. 2

## Data Availability

All data that support the findings of this study are included in this published article and available in the Supporting Information Material of this article.
