# Supplementary material for: SUMOylation of EphB4 enhances its stability in prostate cancer
Source: Br J Cancer. 2026 Apr 15;135(1):48–59. doi: 10.1038/s41416-026-03442-w (PMC13270174; doi:10.1038/s41416-026-03442-w)
Supplement: Supplementary file 1 — Supplementary Fig. Legends [file 41416_2026_3442_MOESM1_ESM.docx]

**Supplementary Figure 1. EphB4 independent SUMO1 signal in 22Rv1 and MCF10A cells.** Confocal imaging of 22Rv1 cells over-expressing the vector only eGFP (green) and stained for SUMO1 (magenta). Cells were treated with clustered Fc protein only (α-Fc) **(A)** or with soluble clustered ephrinB2-Fc **(B)** for 15 min. Nuclei are stained blue with DAPI. **C-D** Confocal imaging of EphB4 over-expressed in MCF10A cells (green) co-localised with of SUMO1 (red). Cells were treated with clustered Fc protein only (α-Fc) **(C)** or with soluble clustered ephrinB2-Fc **(D)** for 15 min. Nuclei are stained with DAPI (blue).

**Supplementary Figure 2.** **EphB4 K616R mRNA is expressed but the protein is not detected. A-B.** Relative EphB4 mRNA (normalised to housekeeping control HMBS) in DU145 cells **(A)** or PC3 cells **(B)** transfected with empty vector (Vo), wildtype EphB4, EphB4 K616R [50]. The expression increases relative to Vo is shown above the bars. Two replicate populations were created for the DU145 cell line (A). **C.** Western analysis of MCF10A cells transiently transfected for 48 h with EphB4 (WT), Vector only (Vo), Lipofectamine (Mock) and EphB4 K616R (K616R). GAPDH was used as a loading control.
