# Supplementary figures and images for: SUMOylation of EphB4 enhances its stability in prostate cancer
Source: Br J Cancer. 2026 Apr 15;135(1):48–59. doi: 10.1038/s41416-026-03442-w (PMC13270174; doi:10.1038/s41416-026-03442-w)

A 22Rv1-VOeGFP +  $\alpha$ -Fc 15 min

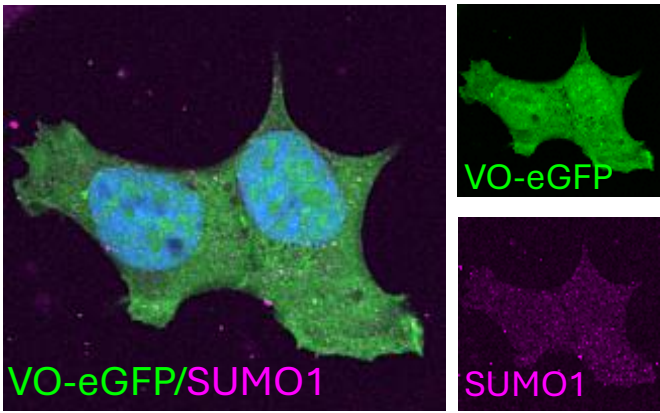

B 22Rv1-VOeGFP + ephrinB2-Fc 15 min

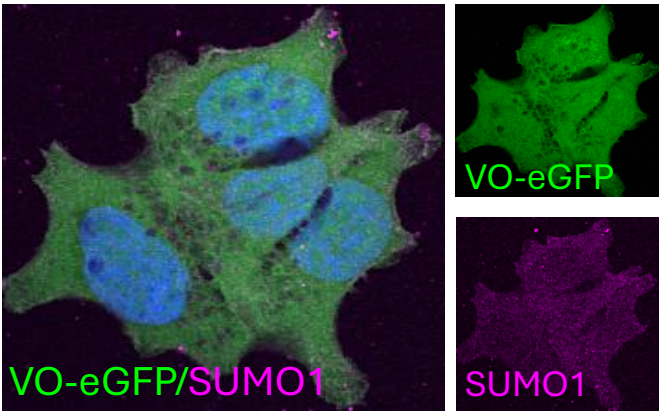

C MCF10A-B4 +  $\alpha$ -Fc 15 min

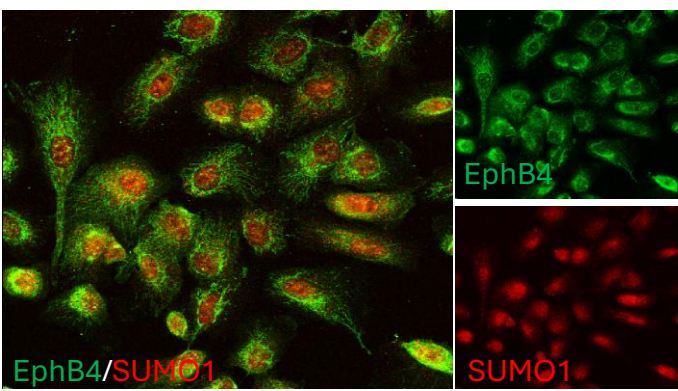

D MCF10A-B4 + EphrinB2-Fc 15 min

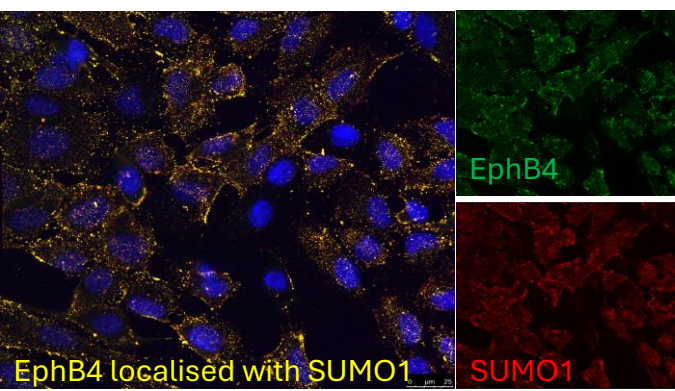

Supplement: Supplementary file 2 — Supplementary Fig. 1 [file 41416_2026_3442_MOESM2_ESM.pdf]

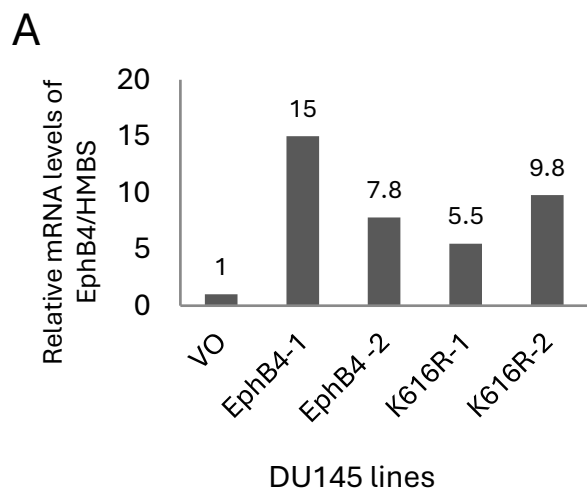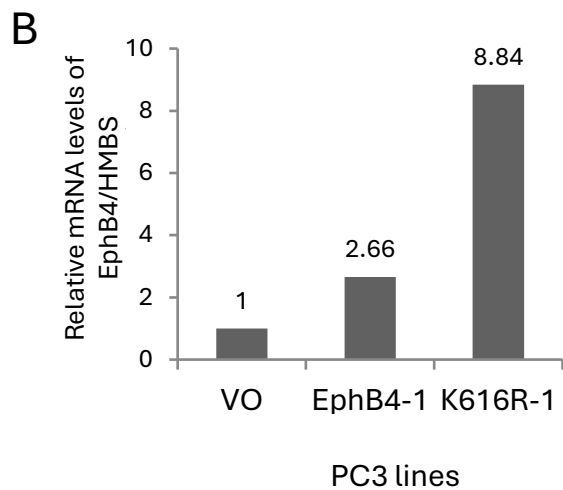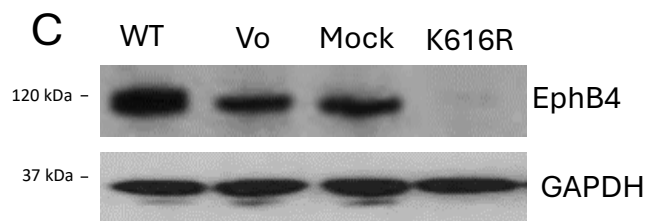

Supplement: Supplementary file 3 — Supplementary Fig. 2 [file 41416_2026_3442_MOESM3_ESM.pdf]
